# PprA Contributes to *Deinococcus radiodurans* Resistance to Nalidixic Acid, Genome Maintenance after DNA Damage and Interacts with Deinococcal Topoisomerases

**DOI:** 10.1371/journal.pone.0085288

**Published:** 2014-01-15

**Authors:** Swathi Kota, Vijaya K. Charaka, Simon Ringgaard, Matthew K. Waldor, Hari S. Misra

**Affiliations:** 1 Molecular Biology Division, Bhabha Atomic Research Centre, Mumbai, India; 2 Division of Infectious Disease, Brigham and Women's Hospital, Department of Microbiology and Immunobiology, Harvard Medical School, and HHMI, Boston, Massachusetts, United States of America; Saint Louis University, United States of America

## Abstract

PprA is known to contribute to *Deinococcus radiodurans*' remarkable capacity to survive a variety of genotoxic assaults. The molecular bases for PprA's role(s) in the maintenance of the damaged *D. radiodurans* genome are incompletely understood, but PprA is thought to promote *D. radiodurans*'s capacity for DSB repair. PprA is found in a multiprotein DNA processing complex along with an ATP type DNA ligase, and the *D. radiodurans* toposiomerase IB (DraTopoIB) as well as other proteins. Here, we show that PprA is a key contributor to *D. radiodurans* resistance to nalidixic acid (Nal), an inhibitor of topoisomerase II. Growth of wild type *D. radiodurans* and a *pprA* mutant were similar in the absence of exogenous genotoxic insults; however, the *pprA* mutant exhibited marked growth delay and a higher frequency of anucleate cells following treatment with DNA-damaging agents. We show that PprA interacts with both DraTopoIB and the Gyrase A subunit (DraGyrA) *in vivo* and that purified PprA enhances DraTopoIB catalysed relaxation of supercoiled DNA. Thus, besides promoting DNA repair, our findings suggest that PprA also contributes to preserving the integrity of the *D. radiodurans* genome following DNA damage by interacting with DNA topoisomerases and by facilitating the actions of DraTopoIB.

## Introduction

*Deinococcus radiodurans* is extremely resistant to many abiotic stresses including ionizing radiation, UV and other DNA damaging agents [1]. After exposure to ordinarily lethal doses of γ radiation, the *D. radiodurans* genome is shattered into numerous double strand breaks (DSBs) and single strand breaks [2]. The genome is subsequently reassembled back to its full length with high fidelity by an extended synthesis dependent strand annealing (ESDSA) DSB repair mechanism [3], [4]. Besides the highly efficient DSB repair mechanism, strong antioxidant mechanisms [5], [6], [7] are also thought to contribute to the remarkable resistance of this bacterium to genotoxic assaults. Another distinctive feature of this bacterium is its highly condensed toroidal genome; however, it remains unclear whether the compactness and shape of the *D. radiodurans* genome contribute to its extraordinary radiation resistance [8], [9]. Also, although *D. radiodurans* encodes both subunits of DNA TopoII and a type IB DNA topoisomerase (DraTopoIB) [10], the roles of these enzymes in *D. radiodurans*' resistance to DNA damage are not known.

A mutation making *D. radiodurans* hypersensitive to radiation and other DNA damaging agents was mapped to a locus named *pprA* (a pleiotropic protein involved in radiation resistance, ORF DR_A0346 in *D. radiodurans* R1) [10]. The PprA protein binds to broken double stranded DNA (dsDNA) and protects it from exonuclease degradation similar to the eukaryotic Ku protein [11]. PprA stimulates both ATP and NAD dependent DNA ligase activities *in vitro* [11]. It was found to be the part of a multiprotein DNA processing complex comprised of 24 proteins including the ATP type DNA ligase (LigB), DraTopoIB (DR_0690) and 11 hypothetical polypeptides of *D. radiodurans* [12]. Subsequently, it was shown that PprA could restore the DNA end joining activity of LigB, which was otherwise inactive in purified form [13], [14]. The involvement of PprA in *Deinococcus* resistance to different DNA damaging agents including radiations and mitomycin C (MMC) has been demonstrated, but not to the Topo II inhibitor nalidixic acid (Nal), which also damages DNA.

DNA topoisomerases are ubiquitous enzymes that help cells to maintain the correct topology of their DNA. Routine cellular processes, including DNA replication, transcription and recombination alter DNA topology, and topoisomerases are essential for restoring proper topology and maintaining genome integrity. Topoisomerases are typically classified into type I (TopoI) and type II (TopoII) based on their substrate preferences (the number of nicks in DNA), subunit structures, and cofactor requirements. Phylogenetically, these enzymes are grouped into TopoIA, IB and IC, and Topo IIA and IIB, respectively [15], [16]. TopoI subtypes are highly divergent in terms of both structure and function [17], [18]. *D. radiodurans* encodes a Topo IB that is structurally similar to the TopoIB of poxviruses [19], and this class of enzymes forms a transient covalent link 3′ of the DNA break. Topo IB can relax both positive and negative superturns *in vitro*. In *Saccharomyces cerevisiae*, genetic analyses suggest that Topo IB plays a major role in DNA replication, transcription and genome structure maintenance, processes which are ordinarily thought to be accomplished by TopoII [20]. Although, DraTopoIB relaxes both negatively and positively supercoiled DNA *in vitro,* its activity is resistant to camptothecin, a compound that inhibits the activity of nuclear encoded TopoIB [19].

Here we report that a *D. radiodurans pprA* mutant is even more sensitive to inhibition of TopoII activity by Nal than to γ radiation. In normal growth conditions, in the absence of exogenous genotoxic insults, the growth of the *pprA* mutant was indistinguisable from that of the wild type. However, after exposure to either Nal or γ radiation, the mutant exhibited marked growth arrest and an elevated fraction of anucleate cells compared to the wild type. In addition, we found that PprA interacts with DraTopoIB and DraGyrA and could enhance the relaxation of supercoiled DNA by recombinant DraTopoIB *in vitro*. Nal treatment reduced the expression of both *dratopoIB* and *dragyrA* genes, particularly in cells lacking PprA. Collectively, our observations suggest that in addition to its known role in enhancing DSB repair, PprA promotes the maintenance and recovery of the damaged *D. radiodurans* genome by interacting with topoisomerases in this bacterium.

## Materials and Methods

### Bacterial strains, plasmids and media

*Deinococcus radiodurans* R1 (ATCC13939) was a gift from Professor J. Ortner, Germany [21] and the *pprA*::cat mutant was a gift from Prof. I. Narumi, JAERI Japan. Strains were maintained in TGY (0.5% Bacto Tryptone, 0.3% Bacto Yeast Extract, 0.1% Glucose) medium at 32°C in the presence of appropriate antibiotics as required. The *E. coli* strain HB101 was used for maintaining cloned genes on plasmids while strain *E. coli* BL21 (DE3) pLysS was used for protein expression. *E. coli* and *D. radiodurans* were grown as batch cultures in LB broth or TGY broth, as required with shaking at 180 rpm. The shuttle expression vector p11559 [22] and its derivative pVHS559 [23] were maintained in *E. coli* strain HB101 in presence of spectinomycin (40 µg/ml), while in *D. radiodurans* these vectors were maintained in presence of spectinomycin (75 µg/ml). All recombinant techniques were as described earlier [24].

### Construction of DraTopoIB expression plasmid

Genomic DNA of *D. radiodurans* R1 was prepared as described in [25]. The 1041 bp coding sequence of DraTopoIB (DR_0690) was PCR amplified from genomic DNA using topoIF (5′ CGCGGATCCATGCCGAGCCGCACCGAA′) and topoIR (5′ CCCAAGCTTTCATTTAGCGCCCGGCC3′) primers containing appropriate restriction enzyme sites at their respective 5′ ends. The PCR product was inserted into the *Bam*HI and *Hind*III sites of the pET28a+ expression vector. The resulting recombinant plasmid, pETtopoIB, was used for the expression of recombinant DraTopoIB in *E. coli*. The PprA expression plasmid pETpprA was constructed as described earlier [26]. For construction of pVHSpprA, the pprA coding sequence was PCR amplified using pprAF (5′ CGCGGTACATATGGTGCTACCCCTGGCCTT 3′) pprAR (5′ CCGCTCGAGTCAGCTCTCGCGCAGGCCGT3′) primers and cloned into the *Nde*I-*Xho*I sites of pVHS559 [23] to yield pVHSpprA.

### Nalidixic acid and γ radiation treatment

Wild type *D. radiodurans*, the *pprA* mutant, and the *pprA* mutant expressing wild type PprA on pVHSpprA under the control of an IPTG inducible promoter were grown in TGY medium and serial dilutions of these cells were spotted on TGY agar plates supplemented with Nal (20 µg/ml). These cells were also exposed to the indicated doses of γ radiation as described earlier [27] at a dose rate of 4.16 kGy/h in Gamma chamber (GC 5000, ^60^Co, Board of Radiation and Isotopes Technology, India). Different dilutions of treated cultures were spotted on TGY agar plates. The plates were incubated at 32°C for 48 h and growth was monitored.

### Microscopy techniques

Fluorescence microscopic studies were carried out as described earlier [23], [28]. Cells were stained with Nile red (4 µg/ml) and 4,6 diamdino2-phenylodine dihydroide chloride (DAPI) (0.2 µg/ml), mounted on 1.0% agarose-coated slides and observed under Axio Imager CM5 microscope. Nile red and DAPI fluorescence was recorded at 516 nm and 350 nm, respectively. Images were analyzed using Axiovision 4.8 software, modified by Image J and Adobe photoshop CS3 software. Cells lacking DAPI stained were counted and statistical analysis was carried out using Prism Software and graphs were plotted.

### Protein purification and topoisomerase assay

Recombinant PprA and DraTopoIB expressed in *E. coli* BL21 (DE3) pLysS from pETpprA and pETtopoIB respectively, were purified from soluble fractions by metal affinity chromatography using NiNTA matrix (Qiagen Inc, Germany) followed by Q-sepharose anion exchange chromatography (GE Healthcare) using protocols as described earlier [29]. The DNA relaxation activity of DraTopoIB was monitored as described in [19]. In brief, 0.5 µM DraTopoIB was incubated in a reaction mixture containing 50 mM TrisHCl (pH 7.6), 100 mM NaCl, 5 mM MgCl2 and 500 ng supercoiled pUC18 DNA, in the presence of increasing concentrations of purified PprA as specified in the figure 4 legend. The reaction was carried out at 37°C for 30 min and quenched by heating at 65°C for 15 min in presence of 0.1%SDS. The products were analysed on 1% agarose gels, stained with ethidium bromide and DNA band intensity was measured densitometrically.

### *In vivo* protein –protein interaction

We used the *Escherichia coli* bacterial two-hybrid system as described in [30] to explore *in vivo* protein-protein interactions between PprA and topoisomerases from *Deinococcus radiodurans*. The *pprA* gene was cloned in both pKNT25 and pUT18 at *BamH*I and *Kpn*I sites to generate pUTpprA and pKNTpprA. The *dratopoIB* gene was also introduced into the *Bam*HI and *Kpn*I sites in these vectors. Similarly, deinococcal DNA gyrase A (*dragyrA*) gene was cloned at *Bam*HI and *Kpn*I in pUT18 to generate pUTgyrA, while DNA gyrase B (*dragyrB*) gene was cloned at *Bam*HI and *Kpn*I sites to generate pKNTgyrB. For evaluating the interaction of PprA with DraTopoIB and DraGyrA and/or DraGyrB, these proteins were co-expressed in *E. coli* strain BTH101 as discussed in [30] and isolated colonies were spotted on LB plate containing 100 µg/ml ampicillin, 50 µg/ml kanamycin, 200 mg/L X-gal and 0.5 mM IPTG and incubated at 30°C overnight and documented as described in [30]. For measuring the levels of β-galactosidase activity in different samples, these cells were grown and beta galactosidase activity was measured using ONPG colour substrate and the enzymatic activity was calculated as given in [31].

### Gene expression studies

For monitoring the levels of expression of *draTopoIB* and *dragyrA* genes, quantitative real-time PCR (RT-PCR) was performed as described previously [32] with the RNA isolated from wild type and mutants grown for 4 h in the presence and absence of Nal (20 µg/ml) and treated with RNase free DNase I (Roche) and subsequently purified by phenol chloroform extraction. First-strand cDNA synthesis was carried out in 20 µl reaction containing 1.5 µg of RNA sample using SuperScript III Reverse Transcriptase kit (Invitrogen, Inc.) mixed with random hexamers as per manufacturer protocol and as described in [32]. RT-PCR was carried out using a Corbett rotor gene 3000 PCR machine with gene specific primers and cDNA as template. The primers used included, TIRTF (5′ CAGAAGTTCCGCTATGTCCA 3′) and TIRTR (5′ GGTGATAGCGGTACTGCA 3′) primers for Topoisomerase IB gene, T2ARTF (5′ GCGATGAACGTCATCGT 3′) and T2ARTR (5′ GTGTAGCGCATGTTCCA 3′) primers for gyrase A subunit of DNA TopoII, and gapF (5′-GAAGGGGCCTCCAAGCACAT-3′) and gapR (5′-TTGTACTTGCCGTTCGCGGCT-3′) for the *dr1343* reference gene. Real-time signal detection of RT-PCR product was done using SyBr green 2X master mix kit (Sigma), as per manufacturer's instructions. Levels of transcripts were estimated and normalized by dividing with the levels of reference gene *dr1343* (Glyceraldehyde 3-phosphate dehydrogenase (GAP) transcript.

### Co-immunoprecipitation

Co-immunoprecipitation of DraTopoIB with PprA protein was carried out by mixing ∼2 mg of protein equivalent cell free extract of cells expressing recombinant (His)-PprA and (His)-DraTopoIB separately and incubating at 4°C for 3 h with gentle mixing. The mixture was further incubated with PprA specific antibody [11] in binding buffer (140 mM NaCl, 8 mM sodium phosphate, 2 mM potassium phosphate and 10 mM KCL, pH 7.4) at 4°C overnight as described in [33] with slow shaking. Subsequently, Protein G agarose beads were added for 3 h with gentle shaking. The antigen-antibody complexes was precipitated by centrifugation at 14,000 g for 5 min, and the pellet was washed with pre-chilled binding buffer 3 times (800 µl each) and eluted with 500 mM NaCl in binding buffer. Eluted proteins were precipitated with 2.5 volume ice-chilled acetone and dissolved in 2× Laemmili buffer. Proteins were separated on 10% SDS-PAGE, transferred to PVDF membrane and western blotting was done with anti-His antibodies to detect both proteins.

Data presented without standard deviations are illustrative of typical experiments, where variation among replicates was less than ∼15%. All experiments were carried out at least three times.

## Results

### PprA promotes *D. radiodurans* resistance to nalidixic acid

**Figure 1:**
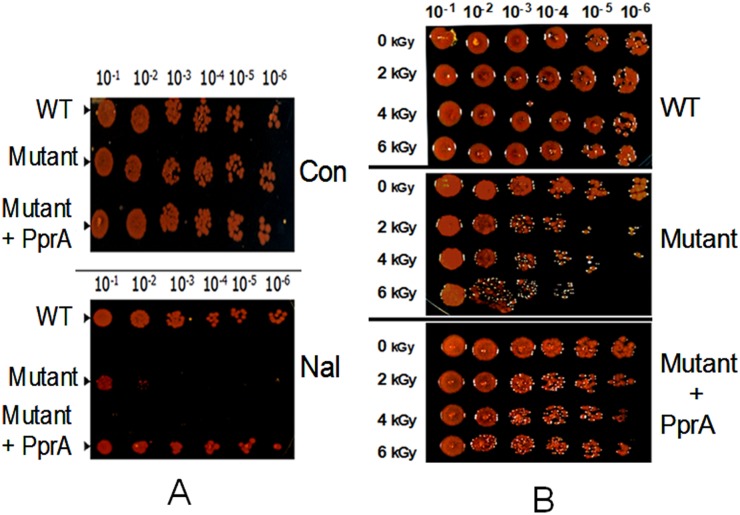
PprA promotes *D radiodurans* resistance to nalidixic acid. Different dilutions of exponentially growing cells of *D. radiodurans* (WT), a *pprA*:*cat* mutant (Mutant) and the mutant expressing PprA *in trans* (Mutant +PprA) were spotted on TGY agar plates in absence (Con) and presence of nalidixic acid (Nal) (20 µg/ml) (A). Similarly, these cells were treated with different doses of γ radiation and different dilutions were spotted on TGY agar plate and growth was monitored (B).

Nalidixic acid (Nal) is a quinolone antibiotic that inhibits DNA topoisomerase II and IV, enzymes that function in a variety of DNA transactions that are important for maintaining the topology of the genome and decatenating intertwined circular chromosomes [16], [34], [35]. Since PprA is known to augment *D. radiodurans*' resistance to DNA-damaging γ radiation, we wondered whether this pleiotropic protein also contributes to the organism's response to treatment with Nal, which also damages DNA [36] and affects genome topology through the inhibition of TopoII activity. A *D. radiodurans* R1 *pprA* mutant (*pprA*::*cat*) proved to be markedly more susceptible to Nal (20 µg/ml) than the isogenic wild type strain (Figure 1A). Nal resistance was restored to the *pprA* mutant by provision of *pprA in trans*, establishing that PprA promotes *D. radiodurans*' resistance to this DNA damaging agent. Notably, the reduction in the survival of the *pprA* mutant following Nal treatment was even more pronounced than that following 6 kGy γ radiation (Figure 1B), a dose that produces nearly 200 DSBs and 3000 single strand breaks in *D. radiodurans* [2]. The larger magnitude of the killing of the *pprA* mutant following Nal treatment raises the possibility that PprA contributes to *D. radiodurans*' survival after DNA damage through mechanisms besides its known role in augmenting DSB repair.

### PprA promotes *D*. *radiodurans*' growth after exposure to γ radiation or nalidixic acid

**Figure 2:**
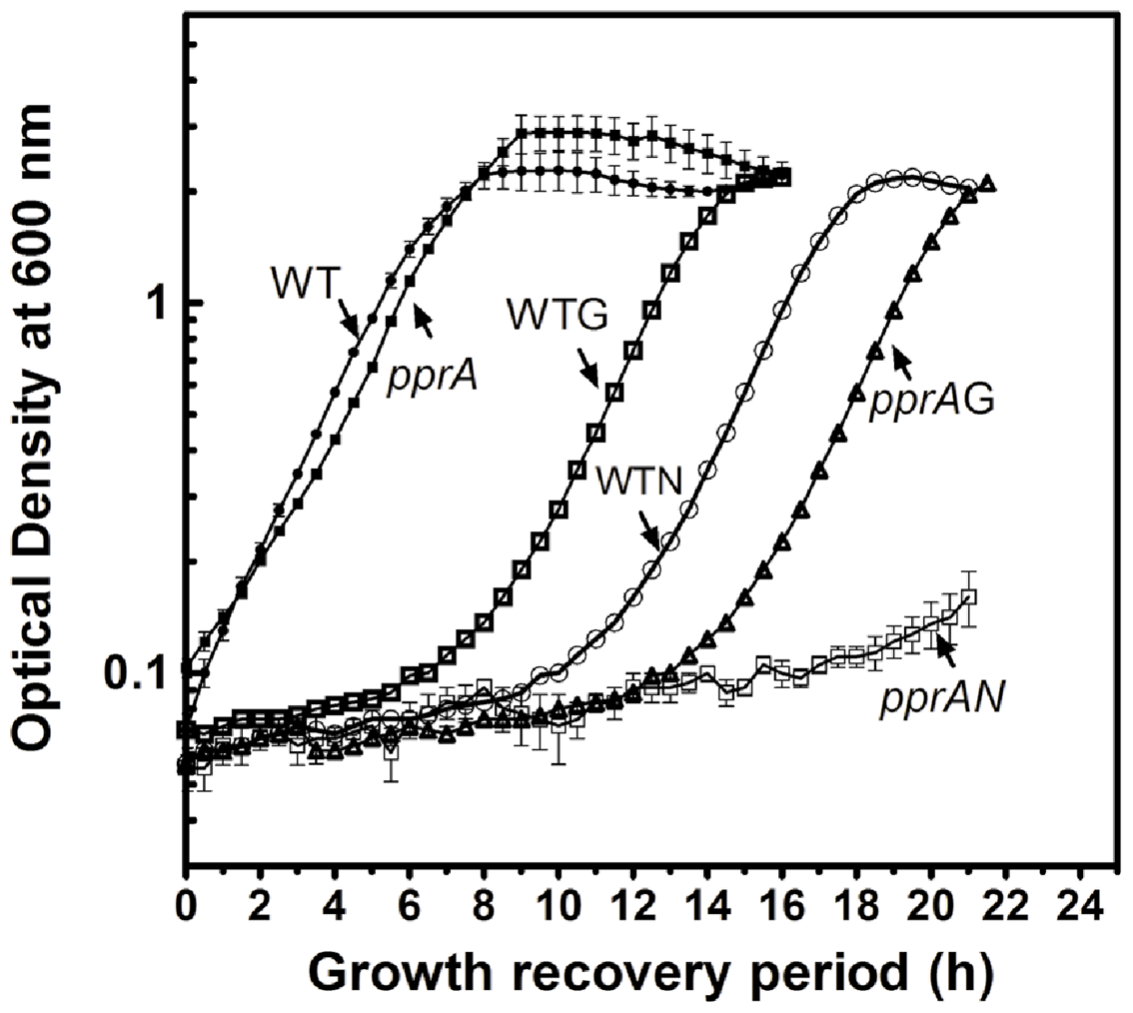
PprA promotes *D. radiodurans* growth following DNA damage. *D. radiodurans* (WT) and a *pprA::cat* mutant (pprA) were treated with nalidixic acid (20 µg/ml) for 2 h (WTN, pprAN) and γ radiation (6 kGy) (WTG, pprAG). These cells were washed and resuspended in fresh TGY medium, and growth at 30°C was monitored as optical density 600 nm.

Genotoxic treatments lead to varied periods of *D. radiodurans* growth arrest during which damaged DNA is repaired and the integrity of its multipartite genome is restored. In wild type cells following exposure to either 6 kGy γ radiation or Nal (20 µg/ml), there is a ∼6 hr and ∼9 hr lag respectively before growth resumes (Figure 2). Inactivation of *pprA* led to prolonged periods of growth arrest after γ radiation treatment (up to ∼12 hr) and especially after Nal treatment (up to ∼18 hr). Notably, PprA does not appear to augment *D*. *radiodurans* growth in the absence of DNA damage, as wild type *D*. *radiodurans* and the *pprA* mutant exhibit very similar growth curves (Figure 2). Collectively, these observations suggest that *pprA* makes a critical contribution to *D*. *radiodurans* recovery and/or growth following damage to its DNA, but it is not required for growth in the absence of exogenous genotoxic insult.

### PprA promotes maintenance of the *D*. *radiodurans* genome after exposure to DNA damage

**Figure 3:**
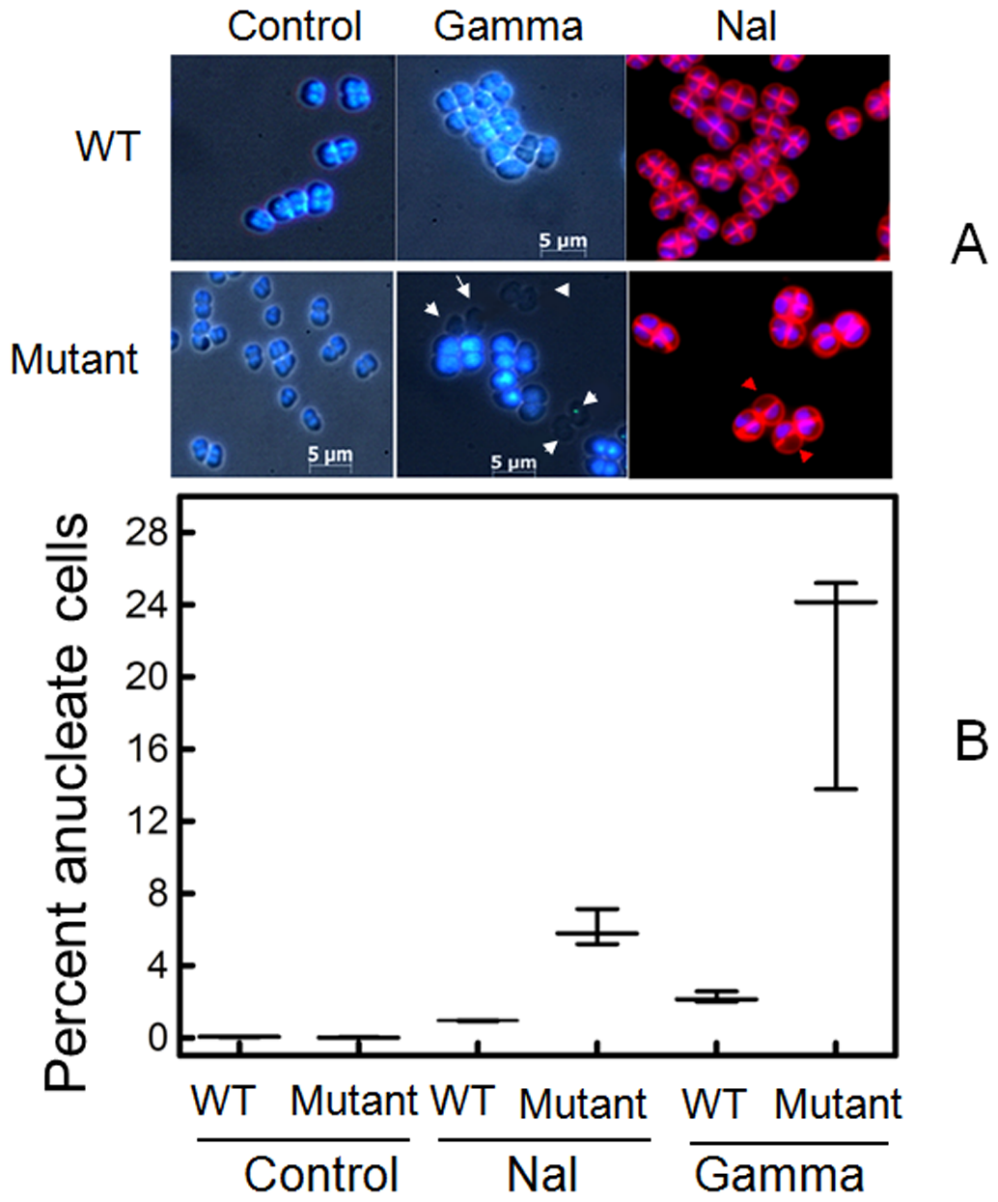
PprA promotes *D. radiodurans* genome maintenance following DNA damage. Both wild type (WT) and *pprA* mutant (Mutant) *D. radiodurans* cells were treated with nalidixic acid (Nal) for 2 h and 6 kGy γ radiation (gamma) and stained with DAPI to detect anucleate cells. Representative micrographs are shown in (A), where anucleate cells, lacking DAPI fluorescence, are indicated with arrows. The percent anucleate cells in each condition (∼500 cells/sample) is plotted in (B).

In order to begin to explore the reasons for the extended delays in the growth of the *pprA* mutant following γ radiation and Nal treatments, we used DAPI staining to assess the state of the *D*. *radiodurans* nucleoid after exposure to these agents. Under normal growth conditions in TGY medium, it was difficult to detect anucleate cells with DAPI staining in both wild type and *pprA* mutant cells (Figure 3). However, there was a marked increase in the frequency of anucleate *pprA* mutant cells following their exposure to Nal (20 µg/ml) for 2 h, or γ radiation (6.0 kGy): 6.7±0.8% and 23.9±1.6% respectively. In contrast, the frequency of anucleate wild type cells increased to only 1.0±0.2% and 2.3±0.2% when wild type cells were subjected to these 2 treatments. Thus, *pprA* appears to specifically promote the integrity of the *D*. *radiodurans* genome after the organism is exposed to exogenous genotoxic conditions and not during routine growth.

### Recombinant PprA interacts with recombinant DraTopoIB and stimulates its activity

**Figure 4:**
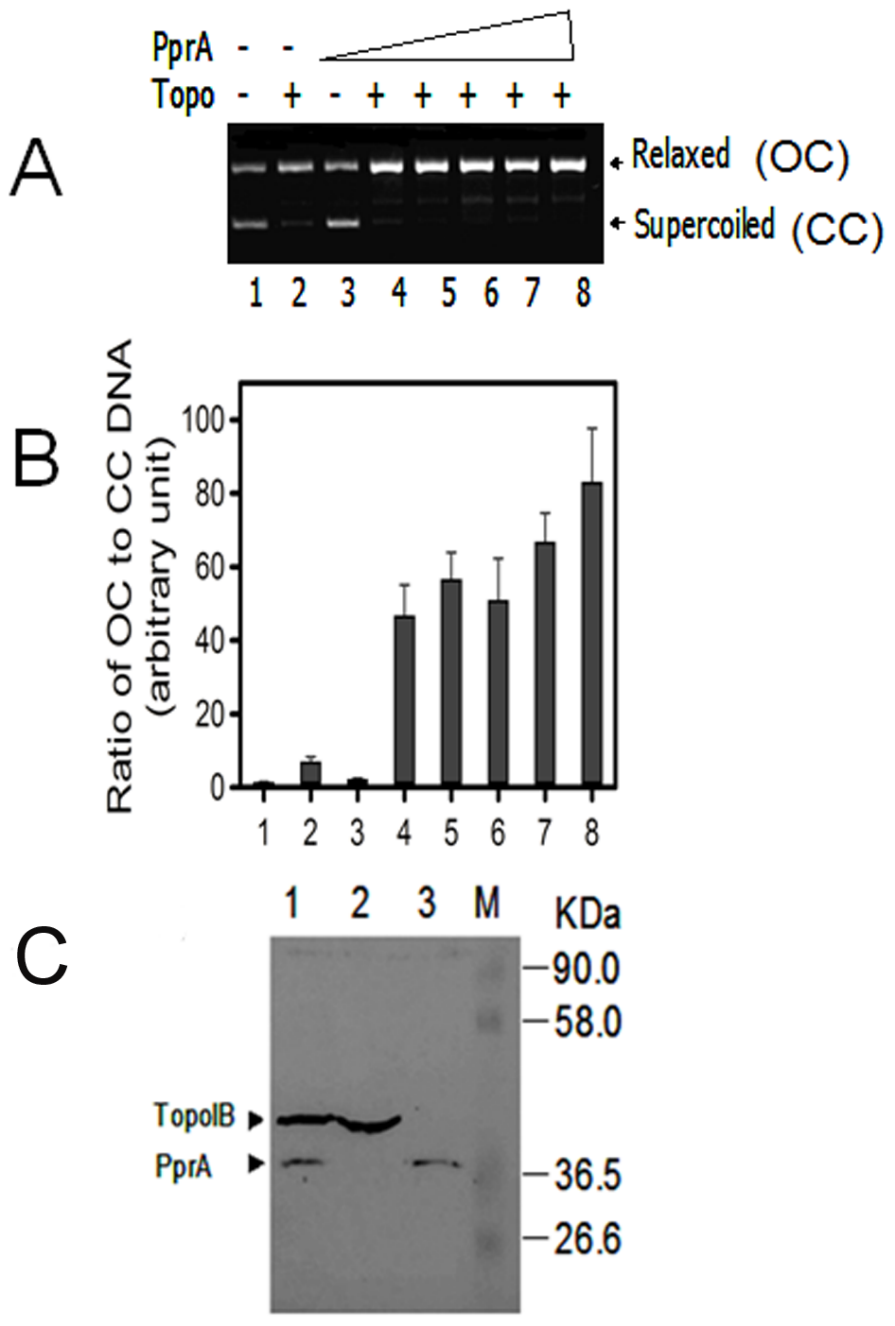
Effect of purified PprA on *D. radiodurans* Type IB topoisomerase activity. Plasmid DNA having both supercoiled (CC) and relaxed (OC) species was incubated with purified recombinant DraTopoIB (Topo) alone and with increasing molar ratio (1, 2, 3, 4 and 5) of purified PprA. Products were analysed on 1% agarose gels (A). Supercoiled (CC) and relaxed (OC) DNA band intensities were quantified and the ratios of OC to CC were plotted (B). In (C), cell free extracts of *E. coli* expressing PprA and DraTopoIB from pETpprA and pETtopoIB plasmids respectively were immunoprecipitated with anti PprA antibodies (1). This immunoprecipitate and extracts of cells expressing pETtopoIB (2) and pETpprA (3) independently were immunoblotted with antibodies against (His)6 tag and the sizes of these immunostained bands were compared to the molecular weight marker (M).

PprA was found to be present along with several other proteins, including LigB (DR_B0100), DncA (DR_2417), a nuclease [29], and DraTopoIB (DR_0690) in a multiprotein ‘DNA-processing complex’ that is thought to play a critical role in this organism's remarkable resistance to γ radiation. Earlier, PprA interaction with LigB (DR_B0100) and T4 DNA ligase and stimulation of ligase activity of these enzymes has been demonstrated [11], [14]. Since DraTopoIB is known to play an essential role in the maintenance of proper DNA topology by relaxing the positive superturns accumulated during DNA replication, transcription, and recombination [19], [35], [37], we speculated that the PprA – topoisomerase interaction might also be important for maintenance/restoration of the integrity of the *D. radiodurans* genome following exposure to genotoxic agents. To begin to explore this idea, we tested whether PprA modulated the activity of purified recombinant DraTopoIB using a supercoiled DNA relaxation assay, where conversion of a supercoiled plasmid DNA (CC) to a relaxed molecule (OC) was monitored. On its own, PprA had no effect on the CC to OC conversion (Figure 4A, lane 3), whereas DraTopoIB alone could mediate the conversion of the CC form to the OC form (Figure 4A, lane 2). However, addition of PprA to DraTopoIB markedly increased the ratio of OC to CC (Figure 4B, lanes 4–8), indicating that PprA enhanced the activity of DraTopoIB. The identity of the DNA band located between the OC and CC forms of the DNA, which increased in abundance as more PprA was added to the reaction is unknown.

Interaction between plasmid-expressed PprA and DraTopoIB in *E. coli* was confirmed with co-immunoprecipitation experiments. When cell free extracts from *E. coli* co-expressing His-PprA and DraTopoIB-His were immunoprecipitated with polyclonal antibodies against PprA, two protein bands with sizes corresponding to DraTopoIB and PprA were detected on immunoblots with monoclonal antibodies against the His tag (Figure 4C lane 1). Mixture of cell free extracts having both DraTopoIB and PprA proteins when processed without PprA antibodies did not yield immunosignals with (His)6 antibodies (data not shown)). Together, these observations suggest that PprA and DraTopoIB do not need additional *D. radiodurans*-specific factors to enable their interaction. Surprisingly, the amount of DraTopoIB was higher than PprA in the immunoprecipitates, raising the possibility that DraTopoIB is present in higher stiochiometric ratio than PprA in the complex.

### PprA influences expression of *dragyrA* and *dratopoIB* and interacts with DraGyrA in addition to DraTopoIB

**Figure 5:**
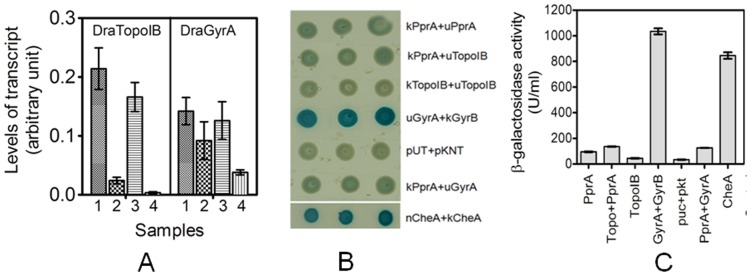
PprA modulates nalidixic acid's effect on gene expression and PprA interactions with DNA topoisomerases. In (A) the effect of nalidixic acid on expression of *dratopoIB* and *dragyrA* genes was monitored by RT-PCR. Total RNA was extracted from both wild type (1, 2) and *pprA* mutant (3, 4) cells grown under normal (1, 3) and in the presence (2, 4) of nalidixic acid (20 µg/ml), and converted to cDNA as described materials and methods. RT-PCR was carried out and levels of expression of both genes were normalized to those of a reference gene, *dr1343,* encoding glyceraldehydes 3-P dehydrogenase. In (B) *E. coli* strain BTH101 cells expressing different proteins on plasmids pUTpprA (uPprA) and pKNTpprA (kPprA), pUTtopoIB (uTopoIB), pKNTtopoIB (kTopoIB), pUTgyrA (uGyrA), pKNTgyrB (kGyrA), pUTcheA (uCheA) and pKNTcheA (kCheA) in different combinations were spotted on LB agar plates supplemented with IPTG and X gal. Plates were incubated at 30°C overnight. Results were compared with *E. coli* strain BTH101 harboring pUT18 and pKNT25 vectors (pUT+pKNT). Similarly in (C) these six strains were induced with 200 µM IPTG for five hours and levels of β -galactosidase activity was monitored on ONPG substrate and blue color product was measured spectrophotometrically. Enzyme activity (U/ml) was calculated as described in materials and methods.

To begin to address how the absence of *pprA* renders *D. radiodurans* extremely sensitive to Nal, we analyzed the effect of Nal on expression of the *dratopoIB* and *dragyrA* genes in the wild type and *pprA* mutant backgrounds. Nal affected expression of both *dratopoIB* and *dragyrA* genes and this effect was more prominent in the absence of PprA (Figure 5A). For example, wild type cells treated with Nal showed ∼6 and ∼1.5 fold reduction in the abundance of *dratopoIB* and *dragyrA* transcripts, respectively (Figure 5A, compare lane 1 and 2). However, in Nal treated *pprA* mutant cells, the abundance of these transcripts decreased by ∼50 fold for *dratopoIB* and ∼3.3 fold for *dragyrA* as compared to the respective untreated controls (Figure 5A, compare lane 1 and 2 with lane 3 and 4), indicating that Nal-induced effects on expression of these genes were amplified in absence of PprA.

We used the bacterial two hybrid (BTH) system to further evaluate PprA interactions with deinococcal gyrase (TopoII) and DraTopoIB. In these experiments, PprA and the subunits of these topoisomerases were introduced into the pUT18 and pKNT25 expression vectors [30] and β-galactosidase expression was monitored as a way to assess protein interactions as described earlier. We used the previously demonstrated CheA-CheA interaction [38] as a positive control for these experiments. Notably co-expression of DraGyrA and DraGyrB yielded as strong a signal as CheA/CheA in this reporter system (Figure 5BC), consistent with the known interactions of these 2 gyrase subunits in other organisms and also validating that the BTH can be used to detect interactions of *D. radiodurans* proteins. Although the signal was not as strong as from the DraGyrA and DraGyrB interaction, co-expression of PprA with either DraTopoIB or DraGyrA also yielded β-galactosidase expression that was greater than the vector controls (pUT18 and pKNT25) (Figure 5BC), strongly suggesting that PprA can interact with both of these topoisomerases. PprA was also found to interact with itself in this system, consistent with our unpublished findings that this protein oligomerizes. Together these observations suggest that PprA interacts with both TopoII and TopoIB in *D. radiodurans*.

## Discussion

Our findings underscore the idea that PprA, a protein identified as important for *D. radiodurans*' remarkable resistance to radiation more than a decade ago [39], has several activities that contribute to the organism's survival in response to genotoxic insults. Previous studies have demonstrated that PprA promotes *D. radiodurans* DSB repair by stimulating the DNA end joining activity of DNA ligases [11], [14]. In addition to PprA's contribution to DNA repair, recent work suggests PprA plays a role in *D. radiodurans* cell division and genome segregation [40], though the molecular bases linking PprA to these cellular processes were not defined. Here, we found that PprA stimulates the DNA relaxation activity of DraTopoIB and contributes to *D. radioduran*'*s* resistance to the TopoII inhibitor Nal and to the maintenance of the damaged genome in this bacterium. Furthermore, we observed that PprA interacts with both types of topoisomerases and it insulated cells from the Nal induced reduction in expression of *dragyrA* as well as *dratopoIB* genes in *D. radiodurans*. Thus, our observations strongly suggest that interactions between PprA and the topoisomerases that control the topology of the *D. radiodurans*' genome play a heretofore-unappreciated role in PprA's contribution to *D. radiodurans*' resistance to genotoxic assaults.

It is tempting to speculate that both Topo II, which is inhibited by Nal, and DraTopoIB, which is stimulated by PprA, contribute to maintaining the integrity of the *D. radiodurans* genome and hence its survival after its DNA has been damaged. While speculative, this model could explain why the *pprA* mutant is particularly sensitive to damage caused by Nal; in this setting, there is little topoisomerase activity because TopoII is inactivated by the antibiotic as well as its level is reduced, and also DraTopoIB expression as well as activity is reduced due to the absence of PprA. Despite the higher frequency of anucleate cells in the *pprA* mutant after γ radiation vs Nal treatment, the latter treatment was even more toxic to *D. radiodurans* than the former (see Figure 1 and Figure 2). The explanation for this discrepancy is not known, but it appears that the net effect of inhibition of both TopoII and DraTopoIB is more toxic to the *D. radiodurans* cell than the extensive DNA damage caused by γ radiation.

Mechanistically, it is hard to visualize how PprA stimulates two distinct DNA metabolic processes *in vivo* (end joining and relaxation). However, we know that a multiprotein complex comprised of LigB, DraTopoIB and PprA also exhibited the capacity for both relaxation of superhelical molecules and DNA end joining activity [12]. These two DNA transactions are differentially regulated by ATP *in solution*. Therefore, we may speculate that PprA has the unique ability to interact with and stimulate other proteins' functions, but protein specific functions depend upon cofactors requirements which may be favorable for one activity and unfavorable for another.

In summary, our findings suggest that PprA contributes to the maintenance of *D. radiodurans* DNA topology, likely through topoisomerases, in addition to its well-known role in DSB repair. Although, PprA interacts with the GyrA subunit of TopoII, it remains to be seen whether PprA modulates TopoII activity. PprA's facilitation of DNA repair and maintenance of DNA topology both promote the integrity of the *D. radiodurans* genome. Future studies can test whether these 2 distinct activities depend on PprA's capacity to preferentially bind DNA containing breaks. Regardless of the mechanism(s), it is important to emphasize that the *pprA* mutant only exhibited detectable phenotypes (growth arrest and increased anucleate cells) following exposure to DNA damaging conditions. Thus, PprA's pleiotropic functions maintaining the integrity of the *D. radiodurans* genome do not appear to be active under normal growth conditions.
